# A Rejoinder to Dr. Hughes

**Published:** 1888-12

**Authors:** 


A REJOINDER TO DR. HUGHES. By Prosper Bender, M. D.

In his address, delivered before the Hahnemann Society of the Boston University of Medicine, Dr. Prosper Bender commented upon the Dr. Hughes' work on "Therapeutics." To this criticism Dr. Hughes made reply in the *New England Medical Gazette*. Dr. Bender now answers. We quote a sentence or two from this able rejoinder, as they give a concise reason why true homœopaths should reject Dr. Hughes' work. On page five, we read:

"In truth, dear Dr. Hughes, you have sought to give your readers an *easy system of Homœopathy*; a dependence upon which will frequently lead to professional failure. Your system is, in a measure, *the old-school generalization*, exempting one from the laborious method of the differentiating of the elements of the case and of drug-action. You overlook the subjective symptoms, the modalities, conditions, etc., which generally enable the Hahnemannian to prescribe successfully." (Italics ours.)

